# Characterization and applications of a Crimean-Congo hemorrhagic fever virus nucleoprotein-specific Affimer: Inhibitory effects in viral replication and development of colorimetric diagnostic tests

**DOI:** 10.1371/journal.pntd.0008364

**Published:** 2020-06-03

**Authors:** Beatriz Álvarez-Rodríguez, Christian Tiede, Alexis C. R. Hoste, Rebecca A. Surtees, Chi H. Trinh, Gillian S. Slack, John Chamberlain, Roger Hewson, Alba Fresco, Patricia Sastre, Darren C. Tomlinson, Paul A. Millner, Thomas A. Edwards, John N. Barr

**Affiliations:** 1 School of Molecular and Cellular Biology, University of Leeds, Leeds, United Kingdom; 2 INGENASA, Inmunología y Genética Aplicada S.A., Madrid, Spain; 3 National Infection Service, Public Health England, Porton Down, Salisbury, United Kingdom; School of Veterinary Medicine University of California Davis, UNITED STATES

## Abstract

Crimean-Congo hemorrhagic fever orthonairovirus (CCHFV) is one of the most widespread medically important arboviruses, causing human infections that result in mortality rates of up to 60%. We describe the selection of a high-affinity small protein (Affimer-NP) that binds specifically to the nucleoprotein (NP) of CCHFV. We demonstrate the interference of Affimer-NP in the RNA-binding function of CCHFV NP using fluorescence anisotropy, and its inhibitory effects on CCHFV gene expression in mammalian cells using a mini-genome system. Solution of the crystallographic structure of the complex formed by these two molecules at 2.84 Å resolution revealed the structural basis for this interference, with the Affimer-NP binding site positioned at the critical NP oligomerization interface. Finally, we validate the *in vitro* application of Affimer-NP for the development of enzyme-linked immunosorbent and lateral flow assays, presenting the first published point-of-care format test able to detect recombinant CCHFV NP in spiked human and animal sera.

## Author summary

Crimean-Congo hemorrhagic fever virus (CCHFV) is one of the most lethal human pathogens in existence. No approved vaccine or therapies exist and rapid diagnosis of CCHFV is a critical aspect of disease management. We describe the selection and characterization of a high affinity non-antibody binding protein, Affimer-NP, that specifically recognises the CCHFV nucleocapsid protein (NP). Affimer-NP interferes with the RNA-binding function of CCHFV NP and inhibits CCHFV gene expression in mammalian cells. Solution of the crystallographic structure of the CCHFV NP/Affimer-NP complex at 2.84 Å resolution revealed the structural basis for this interference, and we validated the *in vitro* application of this novel molecule for the development of ELISA and lateral flow assays, presenting the first published prototype point-of-care test able to detect recombinant CCHFV NP in spiked human and animal sera. These findings present a possible starting point for the future development of anti-viral molecules targeted to CCHFV NP, and diagnostic assays for the detection of CCHFV NP, contributing to the preparedness for potential future outbreak scenarios.

## Introduction

Crimean-Congo hemorrhagic fever orthonairovirus (CCHFV) is an emerging arbovirus that causes serious human disease characterized by an acute febrile illness with frequent progression to hemorrhagic fever [1]. CCHFV outbreaks can result in alarming mortality rates of up to 80% in hospital settings [2]. The incidence of CCHFV-mediated disease closely matches the geographical range of its *Hyalomma* tick host, which is widespread throughout Africa, Asia, and Southern Europe [3]. As such, CCHFV is the most widespread tick-borne virus on earth [3,4].

CCHFV is classified within the *Nairoviridae* family of the order *Bunyavirales* [5]. Its genome comprises three negative sense RNA segments named small (S), medium (M) and large (L), which respectively encode the nucleoprotein (NP), that encapsidates each RNA segment; the glycoprotein precursor, which is post-translationally cleaved to yield envelope glycoproteins Gn and Gc; and the L protein that contains both RNA-dependent RNA polymerase (RdRp) and OTU-like modules, with the latter performing immunomodulatory roles. The RdRp and NP associate with the segments forming ribonucleoproteins (RNPs) [6] that act as templates for all viral RNA synthesis activities. The structure of native viral RNPs has not been elucidated, but crystal structures of recombinant CCHFV NP [7–9] have revealed possible residues involved in RNA-binding and NP-NP interactions, validated using reconstituted mini-genome systems [10].

Due to its high pathogenicity and lack of efficacious treatments, CCHFV is a prioritized pathogen, with an urgent need for accelerated research in disease diagnosis and treatment [11]. Differential diagnosis during the early disease phase is difficult due to associated non-specific febrile symptoms. RT-PCR methods are the current gold standard for detection of CCHFV during the viremic phase, offering sensitive and highly specific results [12]. An antigen-detection ELISA test (VectoCrimea-CHF-antigen, VectorBest) is commercially available, but most available immunoassays are based on detection of CCHFV-specific IgG or IgM [13–17]. Such tests are normally used in animal serosurveys [16,18] or during late phases of disease but, in severe patients cases, the production of antibodies is normally delayed, low, or even absent [19].

Late diagnosis decreases treatment efficacy and increases the risk of fatal outcomes and nosocomial spread [20], making quick and accurate detection of antigen important for disease management. In addition, fatal outcome is associated with higher viral loads [21,22]. For these reasons, the development of a point-of-care test that enables early and rapid detection of CCHFV antigen in remote areas or low-resource settings is an urgent and unmet need [23].

Here, we describe the selection of a high-affinity small protein (Affimer-NP) that binds specifically to CCHFV NP and blocks both its RNA-binding function and ability to support gene expression. The crystal structure of Affimer-NP bound to CCHFV NP revealed the interaction contacts between these two molecules, explaining these interference characteristics. In addition, we validated the use of Affimer-NP for the development of ELISA and lateral flow tests that detected CCHFV NP in human and animal spiked sera.

## Methods

### Production of bunyaviral nucleoproteins

CCHFV, Hazara virus (HAZV), Schmallenberg virus (SBV) and Rift Valley fever virus (RVFV) NPs were expressed in bacteria and purified as previously described [10,24].

### Generation of anti-CCHFV NP IgGs

CCHFV NP was lyophilized using an AdVantage 2.0 Lyophilizer (SP Scientific) and used as immunogen (GenScript). Rabbit polyclonal IgGs were purified by Protein A affinity chromatography.

### Screening for CCHFV NP-binding Affimers

Affimer screening was performed as previously described [25], using CCHFV NP as target. ORFs of unique binders were subcloned into pET11a expression vector for production of the corresponding Affimers appended with an N-terminal 8x his tag and cysteine for downstream functionalization using bacterial expression (*E*. *coli* strain Rosetta 2), and purified using Ni^2+^-NTA affinity chromatography as previously described [26].

### Affimers biotinylation

Immobilized TCEP disulphide reducing gel (Thermo-Fisher) was used to reduce Affimer disulphide bonds according to manufacturer’s instructions. For biotin labelling, 6 μL of biotin maleimide in DMSO (5 mg/mL) were added to reduced Affimers (0.5 mg/mL) and incubated at RT for 2 h. Zeba spin desalting columns (7 kDa MWCO, Thermo-Fisher) were used to remove free biotin.

### Pull-down assays

Dynabeads MyOne Streptavidin T1 (Thermo-Fisher) were blocked overnight with casein blocking buffer (Sigma-Aldrich). After washing, beads were incubated with 2 μg of biotinylated Affimer for 1 h at RT. After further washing, beads were incubated with 10 μg of target molecule and pull-down assays were performed using a KingFisher Flex System (Thermo-Fisher). Samples were heated for 5 min at 95°C, beads pelleted at 13,000 rpm and removed using a magnetic rack.

### ELISA to check biotinylation of Affimers

Briefly, serial dilutions of biotinylated Affimers in PBS were added to Nunc-Immuno MaxiSorp strips (Thermo Scientific) and incubated overnight at 4°C. After washing with PBS-T, 250 μL of blocking buffer (Casein Blocking Buffer 10x, Sigma-Aldrich) were added and strips were incubated at 37°C for 3 h. Strips were washed with PBS-T and streptavidin-HRP in blocking buffer was added. After incubation for 1h at RT on a vibrating platform shaker, wells were washed with PBS-T and 50 μL of TMB (SeramunBlau fast TMB substrate solution, Seramun) were added. Absorbance at 620 nm was measured after 5 min with a plate reader (TECAN).

### Western blotting

Proteins were transferred to polyvinylidene fluoride (PVDF) membranes (Millipore) using a Trans-Blot semi-dry cell (Bio-Rad) in Towbin for 1 h at 15 V. Membranes were blocked overnight at 4°C in 50% Odyssey blocking buffer (LI-COR) in PBS, then incubated with primary antibody (anti-CCHFV NP IgGs/anti-his-tag mouse IgGs Alexa Fluor-647) for 1 h at RT. Membranes were washed and incubated with anti-rabbit-IRDye 800CW (LI-COR). Fluorescent signal was visualized using an Odyssey Sa Infrared Imaging System (LI-COR).

### Surface Plasmon Resonance

Biotinylated Affimers were immobilized on a Sensor Chip SA (GE Healthcare) via streptavidin-biotin interaction using a Biacore 3000 (GE Healthcare). Affimers were diluted in PBS (100 nM) and injected into their respective flow cells at 5 μL/min until surface density reached 100 response units. A flow cell was left empty as reference surface. CCHFV and HAZV NPs diluted in PBS at different concentrations were injected at 20 μL/min for 120 seconds. BIAevaluation software was used for double-referencing analysis. Affinity and kinetic constants were calculated using a Langmuir 1:1 binding model and steady-state affinity models.

### Circular Dichroism

Protein samples were dialyzed in PBS using a Pur-A-Lyzer Dialysis Kit, MWCO 6–8 kDa (Sigma-Aldrich) and a 1:1 molar mixture of CCHFV NP:Affimer-NP was prepared. Samples were transferred to 1 mm path-length quartz cuvettes. A PBS-only sample was included as reference. Thermal melts were performed in a Chirascan Plus Spectometer (Applied Photophysics) monitoring ellipticity at 190–260 nm between 20°C to 90°C at 1°C/min. Ellipticity values (mdeg) were normalized to mean molar ellipticity per residue (MRE). Thermal data was analysed using Global3 (Applied Photophysics), and secondary structure content predicted using BeStSel [14].

### Large scale expression and purification of the Affimer-NP/CCHFV NP complex

Plasmid sequences and detailed cloning strategies are available upon request. Affimer-NP ORF was subcloned into a pETSUMO bacterial expression vector (pETSUMO-Affimer-NP). Rosetta 2 (DE3) *E*. *coli* cells were transformed with pET-SUMO-Affimer-NP and used to inoculate LB medium containing kanamycin (50 mg/mL). Cell cultures were grown at 37°C and induced by addition of Isopropyl β-D-1-thiogalactopyranoside (IPTG) at 500 mM, followed by further shaking at 18°C for 16 h. Cells were pelleted and resuspended in lysis buffer (500 mM NaCl, 50 mM NaH_2_PO_4_ pH 7.4, 20 mM imidazole, 0.1% Triton X-100, 1 mM MgCl_2_, 1 mg/mL lysozyme (Sigma-Aldrich), a complete protease inhibitor cocktail tablet EDTA-free (Roche), 1 U DNase I/1 L culture, 1 U RNase). Cells were sonicated using a Soniprep 150, after which soluble and insoluble fractions were separated by centrifugation at 45,000 xg for 30 min.

Purification involved a first column affinity chromatography step using Super Ni^2+^-NTA (Generon) equilibrated with binding buffer (500 mM NaCl, 50 mM NaH_2_PO_4_ pH 7.4, 20 mM imidazole). Soluble bacterial lysate was applied to column, washed with binding buffer with increasing concentrations of imidazole, after which 6xhis-SUMO-Affimer-NP was eluted with elution buffer (500 mM NaCl, 50 mM NaH_2_PO_4_ pH 7.4, and 300 mM imidazole). The elution fractions were dialyzed to remove excess imidazole and a total of 0.5 mg of 6xhis-SUMO protease Ulp1 produced in-house was added for every 10 mg of eluted 6xhis-SUMO-Affimer-NP. The mixture was transferred to 10 kDa MWCO dialysis tubing (Thermo Scientific) and dialyzed for 16 hr at 4°C in dialysis buffer (500 mM NaCl, 50 mM NaH_2_PO_4_ pH 7.4). The dialyzed protein was subjected to a second Ni^2+^-NTA affinity chromatography step, and the flow-through containing native Affimer-NP was collected and concentrated to 5 mL using centrifugal filters (Amicon Ultra 15 10 kDa MWCO regenerated cellulose). The sample was injected into an equilibrated HiLoad 26/600 Superdex 75 pg (GE Healthcare) column using an Akta prime at 0.5 mL/min at 4°C and 3 mL fractions were collected, after which the purity of Affimer-NP in eluted fractions was analyzed by SDS-PAGE and Coomassie staining. Pure Affimer-NP was then concentrated using centrifugal concentrators, as above.

For complex purification, concentrated Affimer-NP and CCHFV NP were mixed and injected onto an equilibrated HiLoad 26/600 Superdex 75 pg (GE Healthcare) column using an Akta prime at 0.5 mL/min. Size exclusion chromatography was performed at 4°C and 3 mL fractions were collected. Purity of the complex was analyzed by SDS-PAGE followed by Coomassie staining and then concentrated using Amicon Ultra 15 Ultracel 10 kDa MWCO Regenerated Cellulose concentrators.

### Crystallization, Data Collection, and Refinement

Affimer-NP/CCHFV NP complex was concentrated to 10 mg/mL and crystallized at RT from a 1:1 ratio of protein:reservoir solution (0.2 M NH_4_CH_3_CO_2_, 0.1 M CH_3_COONa 4.6 pH, 30% PEG 4K). For vitrification, crystals were transferred to mother liquor containing 25% glycerol. Diffraction data were collected at DLS beamline I04, and structure of the complex solved at 2.84 Å by molecular replacement using published models of the Adhiron scaffold (PDB:4n6u) and CCHFV NP (PDB:4akl) with Phaser. Interface residues between Affimer-NP and CCHFV NP were predicted using Chimera and PDBePISA.

### Fluorescence anisotropy

For direct binding assays, CCHFV NP was serially-diluted using RNA-binding buffer (100 mM NaCl, 10 mM tris-HCl 7.5, 0.01% Triton X-100) in a black 384-well optiplate (Perkin-Elmer). 3′-Fluorescein RNAs were added, obtaining a final concentration of 2.5 nM FI-RNA/well. For competition assays, Affimers were serially-diluted using RNA-binding buffer. 3′-Fl RNAs mixed with CCHFV NP were added to each well, obtaining a final concentration of 230 nM CCHFV NP and 2.5 nM FI-RNA per well. Reactions were incubated at RT for 30 min and fluorescence polarization measured using a Spark 10M microplate reader (Tecan) with excitation and emission filters at 485 nm and 535 nm, respectively. Nonlinear regression curves were fitted using GraphPad to obtain K_D_ and IC_50_ values.

### CCHFV mini-genome

Detailed plasmid sequences are available upon request. Briefly, the CCHFV S-segment replicon was generated using strain Baghdad-12 (GenBank: AJ538196.1) as reference. The S-segment cDNA was incorporated within a plasmid vector flanked by bacteriophage T7 RNA polymerase promoter (T7POL) and hepatitis delta virus ribozyme, and orientated for expression of viral sense RNA. The S-segment NP open reading frame (ORF) was replaced by a cDNA representing the eGFP ORF to generate pC-SsegUTRs-GFP. Support plasmids (pC-NP and pC-L) expressing Baghdad-12 CCHFV NP and L proteins were generated by inserting the corresponding cDNA sequences (GeneBank: CAD61342.1 and AY947890.1) downstream of T7POL and a cDNA representing the encephalomyocarditis virus (ECMV) internal ribosome entry site. The Affimer-NP ORF fused to the RFP ORF was subcloned into a protein expression vector behind the CMV promoter (pMAX-Affimer-NP-RFP). A negative control plasmid containing the ORF of a yeast SUMO-specific Affimer fused to RFP was also generated (pMAX-Affimer-YS-RFP). Sub-confluent BSRT7 cells in a 12-well plate were transfected with 450 ng pC-L, 150 ng pC-NP, 150 ng pC-SsegUTRs-GFP and increasing concentrations of pMAX-Affimer-NP-RFP or pMAX-Affimer-YS-RFP using TransIT-LT1 (Mirus Bio) as transfection reagent in a 2.5:1 ratio of TransIT-LT1:DNA (μL:μg). Green/red fluorescence were quantified using an IncuCyte Live-Cell analysis system with IncuCyte ZOOM software 2018.

### ELISA for detection of CCHFV NP

High-binding 96-well plates (Sigma-Aldrich) were coated overnight with 1 μg of Affimer-NP in 100 μL carbonate buffer, pH 9.6. Plates were blocked with ELISA stabilizing solution (Surmodics) for 1h. After washing with PBS-T, plates were incubated with 100 μL of spiked sera for 1h, followed by primary antibody (anti-CCHFV NP IgGs). Plates were washed and incubated with 100 μL of goat anti-rabbit IgGs-HRP (Sigma-Aldrich) for 1h, followed by 100 μL of 3,3’,5,5’-tetramethylbenzidine (TMB) Enhanced K-Blue substrate (Neogen) for 10 min. Reaction was stopped with 100 μL of 0.5 M sulfuric acid and absorbance (450 nm) measured with a SpectraMax M5 (Molecular Devices) plate reader.

### Lateral flow assay for detection of CCHFV NP

#### Functionalization of beads

For the test line, anti-CCHFV NP IgGs were conjugated to 300 nm red latex particles (Ikerlat) and for the control line, BSA-biotin was conjugated to 260 nm blue latex particles (Ikerlat). Beads were washed in MES 10 mM pH 6 and their size was measured by dynamic light scattering (DLS) using a Zetasizer Nano S system (Malvern). Beads were activated using EDC (1-ethyl-3-(3-dimethylaminopropyl) carbodiimide hydrochloride) and NHS (N-hydroxysuccinimide) and added to the protein of interest. After incubation for 2 h, 500 mM imidazole was added, and beads were washed with 0.1% Tween-20. Blue and red functionalized beads were mixed, diluted in tris-HCl 25 mM, pH 9.5 and dispensed onto a rayon conjugate pad 25-mm (Operon) using a Matrix 1600 dispensing module (Kinematic Automation). Conjugate pad was incubated 30 min in a MMUFE 500 oven (Memmert) at 45°C.

#### Membrane dispensation

For the test line, Affimer-NP was diluted in tris-HCl 20 mM pH 8.5, containing 5.0% sucrose and 0.095% sodium azide. Anti-biotin IgG for the control line was diluted (1 mg/mL) in the same buffer (pH 7.5). Both reagents were dispensed in two parallel lines on nitrocellulose membrane at 1 μL/cm. Dispensed membranes were dried for 5 min at 45°C.

#### LFA assembly

A master card was assembled on a 72-mm backing card (Kenosha) as follows: HiFlow Plus Nitrocellulose Membrane (HF120) (Millipore), the conjugate pad (Operon) and a 25-mm absorbent pad (Ahlstrom) were pasted and covered with a 65-mm transparent adhesive film (Lohmann). The master card was cut to 4.2-mm width strips using a Matrix 2360 machine (Kinematic Automation).

#### LFA sample testing

Sample (20 μL) and buffer (100 μL) were added sequentially, and results annotated after 10 min., with quantification using a ESE Quant Lateral Flow reader (Qiagen) and Lateral Flow Studio Software.

## Results

### Selection of CCHFV NP-specific Affimers

The goal of this study was to identify, purify and characterize Affimer binding reagents specific for CCHFV NP, and to evaluate their potential for use as specific binding molecules in diagnostic tools as well as in interfering with critical CCHFV activities. Affimer affinity reagents are a class of non-antibody binding proteins based on a phytocystatin consensus sequence that scaffolds two variable peptide regions that represent target recognition sites. They can be selected against diverse targets providing high-affinity binding molecules that mimic the molecular recognition of antibodies. Large bacteriophage (‘phage display) libraries are used for their selection and they can be produced easily and economically by recombinant means in bacterial expression systems [26,27]. Recombinant CCHFV NP was used as target molecule for Affimer screening, as previously described [25]. Twenty-four Affimers were isolated and sequenced resulting in 7 unique binders (Affimers 1, 5, 6, 8, 9, 22 and 24, S1A Fig). After bacterial expression and purification using Ni^2+^-NTA affinity chromatography, Affimers were biotinylated using a biotin maleimide moiety, as was a myoglobin-specific Affimer (Affimer-myo), as a negative control. Presence of biotin was analyzed by ELISA using streptavidin-HRP (S1B Fig).

### Affimer-NP binds specifically to CCHFV NP

**Fig 1:**
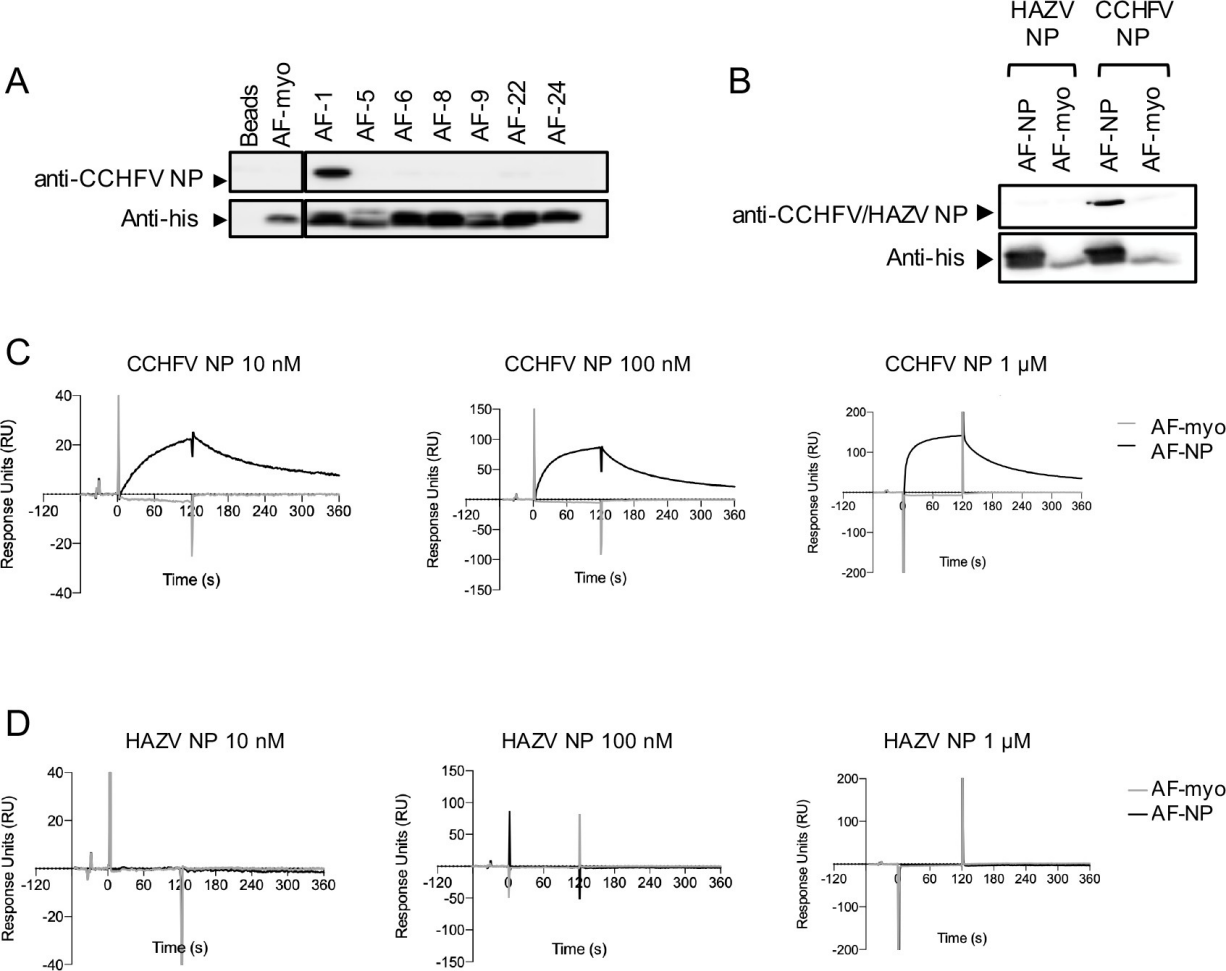
Specificity and affinity of Affimer-NP. (A) Western blot analysis of a pull-down experiment using Affimers as capture molecules and CCHFV NP as target. (B) Western blot analysis of a pull-down experiment using either HAZV or CCHFV NP as target. (C, D) SPR analysis of binding between Affimers and ten-fold dilutions of CCHFV (C) or HAZV NP (D).

The ability of the Affimers to bind CCHFV NP was first assessed by pull-down affinity-precipitation (AP) assays. Briefly, streptavidin-conjugated beads were coated with the 8 biotinylated Affimers and incubated with CCHFV NP. After washing, samples were analyzed by SDS-PAGE and western blotting using anti-CCHFV NP IgGs and an anti-his-tag antibody. Only Affimer-1 showed clear binding to CCHFV NP. All others showed no binding, including the negative control Affimer (Affimer-myo), and the beads-only control (Fig 1A).

Affimer-1 (named Affimer-NP) was chosen as the CCHFV NP binder of interest. To determine the specificity of Affimer-NP for CCHFV NP, additional AP assays were performed using CCHFV NP and NP from the closely-related Hazara virus (HAZV) as target molecules. HAZV forms part of the CCHFV serogroup [28], their NPs share 59% amino acid identity [24] and their crystal structures very closely superpose with an RMSD = 0.70 Å [24] within the respective globular domains. HAZV NP was produced, and AP assays were analyzed by western blotting using the primary antibodies described above (S2 Fig). Affimer-NP showed binding to CCHFV NP but not to HAZV NP, and the negative control Affimer-myo showed no binding to either NP (Fig 1B). These results demonstrate that Affimer-NP selectively recognizes CCHFV NP, showing no cross-reactivity with HAZV NP, despite their close structural similarity.

### Affimer-NP binds CCHFV NP with high affinity

To determine the binding kinetics and affinity of Affimer-NP for CCHFV NP, surface plasmon resonance (SPR) was performed, along with Affimer-myo and HAZV NP as negative controls. Different cells of a streptavidin sensor chip were coated with biotinylated Affimer-NP and Affimer-myo, and ten-fold dilutions of CCHFV or HAZV NP were injected into each cell. CCHFV NP sensograms showed binding to Affimer-NP but not to Affimer-myo (Fig 1C) and conversely, HAZV NP sensograms showed no binding to either Affimer-NP nor Affimer-myo (Fig 1D). CCHFV NP sensograms were fitted to a Langmuir 1:1 binding model and steady state affinity models. The most accurate fitting corresponded to the 10 nM dilution (S3 Fig), presenting the lowest Chi^2^ values relative to the response units of the sensogram (Chi^2^ = 0.104). The fitted curve reveals a low nanomolar K_D_ (K_D_ = 5.69 nM), demonstrating a high affinity interaction between Affimer-NP and CCHFV NP with a fast rate association (K_on_ = 1.17 x 10^6^ M^-1^ s^-1^) and a slow rate dissociation (K_off_ = 6.67 x 10^−3^ s^-1^).

### Secondary structure and thermostability of the Affimer-NP/CCHFV NP complex

**Fig 2:**
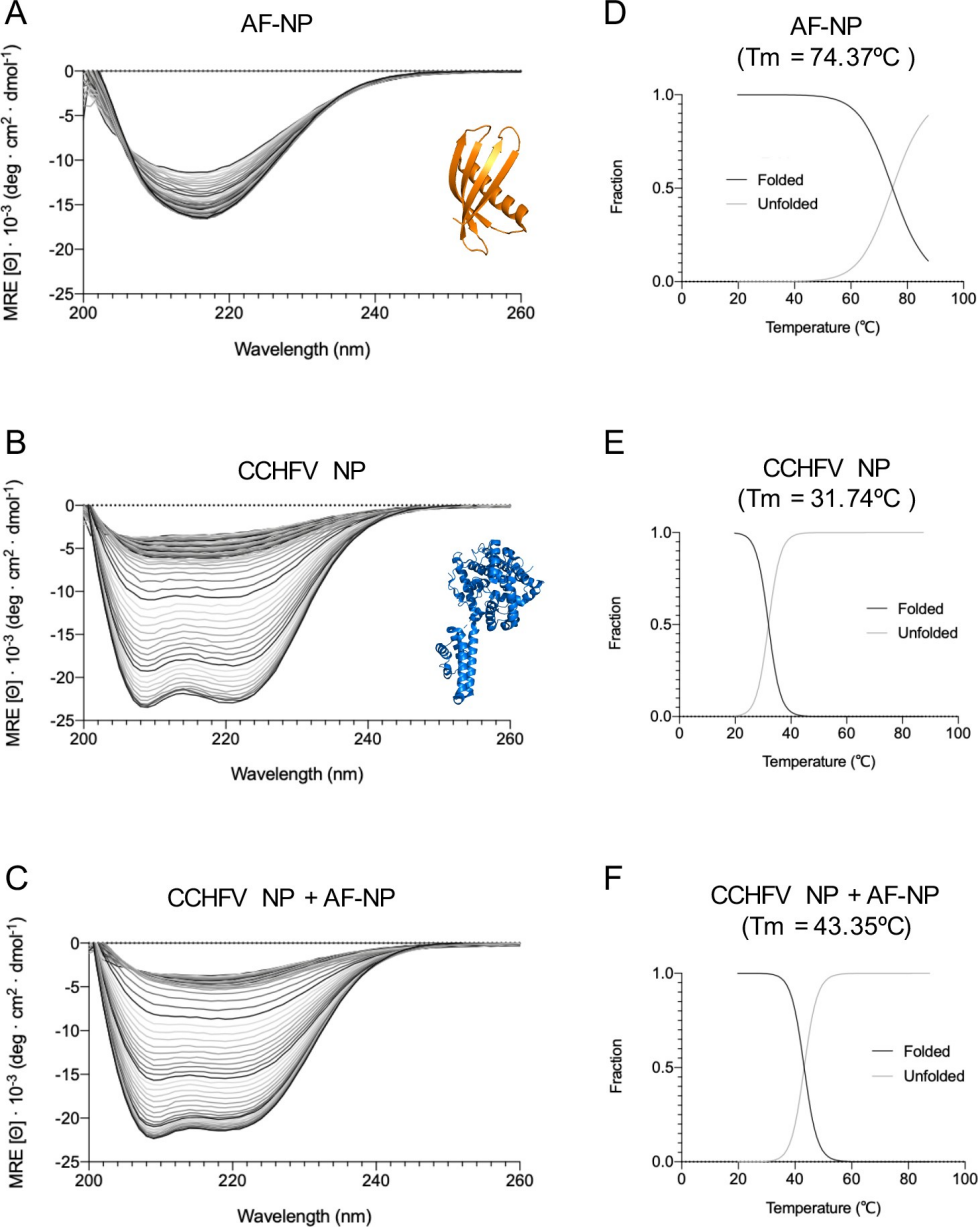
CD analyses of Affimer-NP, CCHFV NP and Affimer-NP/CCHFV NP complex. (A, B, C) Curves representing ellipticity across the far UV spectra of CCHFV NP (A), Affimer-NP (B) or a 1:1 mixture of both (C) at 20ºC-90ºC. Crystal structure of Affimer scaffold (PDB:4n6u) is represented in orange and CCHFV NP (PDB:4akl) in blue. (D, E, F) Curves representing folded/unfolded fractions and melting temperatures (Tm) of Affimer-NP (D), CCHFV NP (E) and their mixture (F).

To better understand the thermal stability of Affimer NP both alone and in complex with CCHFV NP, we next used circular dichroism (CD) to analyze the secondary structure of Affimer-NP, CCHFV NP and a 1:1 molar mix of both, in response to changing temperature. CD spectra were measured between 190–260 nm at temperatures between 20°C to 90°C and secondary structure content was predicted using the online prediction tool BeStSel [29]. Affimer-NP spectra presented a single minimum at 218 nm, indicating a predominance of beta-sheets (Fig 2A), in agreement with the secondary structure prediction software (S4A Fig), and consistent with published scaffold structures [26] (PDB:4n6u, Fig 2A). The melting temperature (Tm) obtained for Affimer-NP was 74.37°C (Fig 2D), also previously observed [26]. CCHFV NP spectra showed two minima at 208 and 222 nm (Fig 2B), which indicates predominance of alpha-helices, also in concordance with both secondary structure prediction (S4B Fig) and published CCHFV NP structures [7] (PDB:4akl, Fig 2B). The Tm obtained for CCHFV NP was 31.74ºC (Fig 2E), also in agreement with published data [24]. The CD spectra of the CCHFV NP/Affimer-NP mixture showed two minima at 208 and 222 nm, indicating a predominance of alpha-helices (Fig 2C; S4C Fig) as expected due to a greater global contribution of CCHFV NP alpha-helices relative to Affimer-NP beta-sheets. The Tm obtained for the mixture (43.35°C, Fig 2F) was higher than the Tm of CCHFV NP alone, indicating that Affimer-NP delayed the melting of alpha-helical structures, predominantly corresponding to CCHFV NP (S4D Fig). Taken together, these CD analyses suggest that Affimer-NP induces thermostabilization of CCHFV NP.

### Affimer-NP competes with the RNA-binding of CCHFV NP

**Fig 3:**
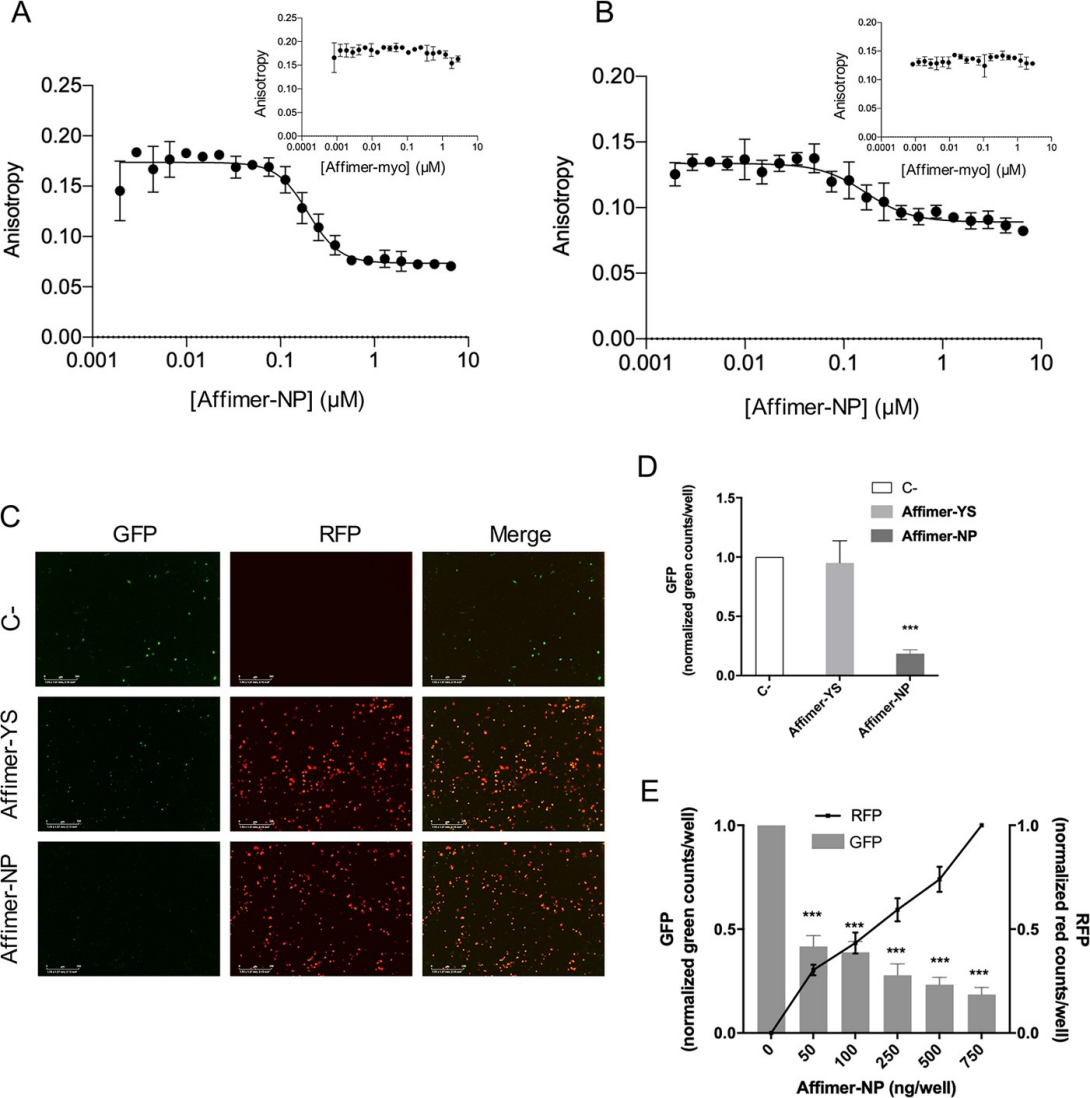
Inhibitory effects of Affimer-NP. (A, B) FA analysis of RNA-binding interference of Affimer-NP using 27mer (A) or 48mer (B) RNA molecules. Top-right graphs represent negative control experiments using Affimer-Myo. (C) IncuCyte pictures taken 72h post-co-transfection of CCHFV mini-genome components plus 750 ng of Affimer-NP-RFP, Affimer-YS-RFP or no Affimer (C-). GFP signal corresponds to CCHFV-specific gene expression and RFP signal corresponds to Affimers-RFP. Scale bar = 300 μm. (D) GFP reporter signal 72h post-co-transfection of CCHFV mini-genome components with 750 ng of Affimer-NP, Affimer-YS or no Affimer (C-). (E) GFP and RFP signal 72h post-co-transfection of CCHFV mini-genome components with different amounts of Affimer-NP-RFP. Data are presented as mean ±SD (n = 3). Statistical analysis was performed using a paired t-test (ns = non-significant, *** = p<0.001).

We next examined whether Affimer-NP may hold the potential to disrupt native CCHFV NP functions, one of which is RNA binding, required during the CCHFV replication cycle for genome encapsidation. The RNA binding ability of CCHFV NP was assessed using fluorescence anisotropy (FA), which measures the tumbling rate of a fluorescently-labelled RNA in solution that is reduced upon interaction with a ligand, in this case CCHFV NP. FA assays were performed to assess whether Affimer-NP interferes with the RNA-binding function of CCHFV NP using two fluorescein-labelled synthetic RNA oligos (27 and 48-mer RNAs). After analyzing the RNA-binding affinity of CCHFV NP to the RNAs *in vitro* and defining the K_D_ of the interaction (K_D 27mer_ = 0.190 μM ± 0.0062, K_D 48mer_ = 0.230 μM ± 0.008, S5 Fig), a competitive assay was performed using Affimer-NP and the negative control Affimer-myo. Affimer-NP showed interference in the RNA-binding of CCHFV NP with IC_50_ values of IC_50 27mer_ = 0.197 ± 0.0084 μM and IC_50 48mer_ = 0.167 ± 0.0046 μM, respectively (Fig 3A and 3B), whereas in contrast, the negative control Affimer-myo, showed no such interference. Taken together, these results show that Affimer-NP specifically-inhibits the RNA binding ability of CCHFV NP.

### Affimer-NP interferes with CCHFV gene expression

The competition of Affimer-NP in the RNA-binding activity of CCHFV NP, which is critical for formation of CCHFV RNA segments, raised the possibility that Affimer-NP would also inhibit CCHFV gene expression. To test this hypothesis, a CCHFV mini-genome system that uses eGFP as reporter of gene expression was established. Plasmids encoding CCHFV mini-genome components were transfected into mammalian-origin BSRT7 cells along with different concentrations of plasmids (50, 100, 250, 500 and 750 ng) expressing Affimer-NP ORF fused to RFP (pMAX-AF-NP-RFP) or a negative control Affimer in which the Affimer-NP sequence was exchanged for a yeast SUMO-specific Affimer (pMAX-AF-YS-RFP). *In vivo* green and red fluorescence signals were monitored using an IncuCyte live cell imaging system, corresponding to CCHFV specific gene expression and the Affimers, respectively. Co-transfection of the replicon with 750 ng of the control Affimer-YS-RFP plasmid resulted in unchanged eGFP reporter signal (Fig 3C and 3D), whereas increasing amounts of Affimer-NP-RFP correlated with a statistically significant decrease in reporter signal (Fig 3D and 3E). These results illustrate a specific and dose-dependent interference of Affimer-NP in CCHFV-specific gene expression.

### Tertiary structure analysis of the Affimer-NP/CCHFV NP complex

To further characterize the interaction between Affimer-NP and CCHFV NP and determine the molecular basis for the interference of Affimer-NP with CCHFV NP RNA-binding and gene expression functions, we solved the high-resolution crystal structure of the complex. Due to the flexibility of the terminal 8xhis and the possibility that the terminal cysteines may form disulphide bonds, Affimer-NP was expressed with N-terminal 6xhis and SUMO Smt3 tags. After initial purification using affinity chromatography, the 6xhis-yeast SUMO tags were removed using ULP1 SUMO protease, and Affimer-NP was further purified using sequential affinity and size exclusion chromatography steps (S6 Fig).

**Fig 4:**
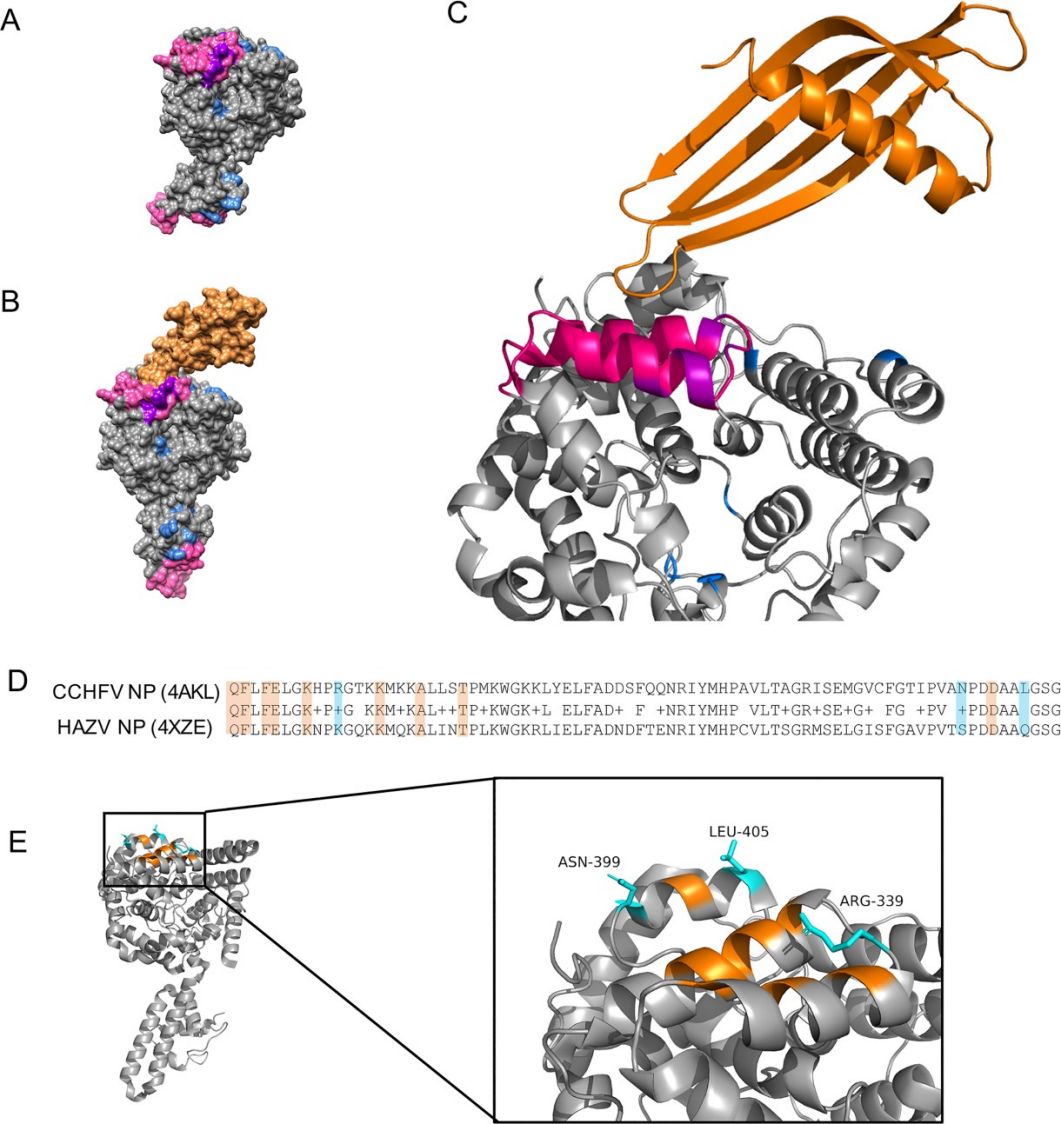
Tertiary structure of Affimer-NP/CCHFV NP complex. (A) Crystal structure of CCHFV NP (PDB:4akl). Residues involved in RNA-binding, monomer-monomer interactions, or both are highlighted in blue, pink and purple, respectively. (B) Crystal structure of Affimer-NP (represented in orange) bound to CCHFV NP. (C) Close-up of Affimer-NP/CCHFV NP interaction. (D) Alignment of CCHFV NP (residues 329–407, PDB:4akl) and HAZV NP (PDB:4xze). NP residues comprising the Affimer-NP variable loop contacts are highlighted in orange, when conserved in both CCHFV and HAZV NPs, or in cyan if not. (E) Close-up of CCHFV NP residues involved in the interaction with Affimer-NP loops. Non-conserved residues (cyan) are labeled.

Affimer-NP and CCHFV NP were mixed and the complex purified by size exclusion chromatography (S6 Fig). The complex was concentrated and crystallized, and the structure was solved at 2.84 Å resolution using Phaser and PDB models 4n6u (Affimer scaffold, Fig 2A) and 4akl (CCHFV NP, Fig 2B) for molecular replacement. Two Affimer-NP/CCHFV NP complexes were elucidated per asymmetric unit. The structure shows the variable loops of Affimer-NP bound to the globular domain of CCHFV NP, mainly interacting with the residues Q329, F330, F332, E333, K336, R339, K343, A347, T351, N399, D402 and L405 (Fig 4B and 4C).

As previously published structures show, CCHFV NP contains two major domains: a globular core and an extended arm [7–9]. Although no crystal structure of CCHFV NP in complex with RNA has been solved to date, analysis of its electrostatic surface potential reveals a continuous positively charged region, likely the binding site for RNA, comprising residues R45, K72, S149, H197, K222, R225, K237, Q303, K336, R339, K342, K343, K345, R372, and K462 [30] (highlighted in blue in Fig 4A–4C). The variable loops of Affimer-NP show interaction contacts with three of these residues (K336, R339 and K343), plausibly explaining the observed disruption of the NP-RNA interaction, described above.

Structural analyses of CCHFV NP also suggest that its oligomerization plays a critical role in RNA-binding, and together these two activities are required for RNP formation and thus gene expression. CCHFV NP exists as a monomer in the absence of RNA, but different oligomeric forms have been described when bound to RNA of the expression host. The interaction between monomers involves residues 320–354 within the head domain of one molecule and residues 210–219 and 260–272 of the arm of the adjacent molecule [9] (highlighted in pink in Fig 4A–4C). The residues of the globular domain that interact with the variable loops of Affimer-NP all lie between NP residues 320–354, suggesting a potential interference of Affimer-NP in CCHFV NP oligomerization. As both oligomerization and RNA-binding are required for assembly of functional CCHFV RNPs, the location of the Affimer-NP binding site also offers a likely explanation for its ability to disrupt CCHFV mini-genome activity.

The specificity of Affimer-NP for CCHFV NP, showing no cross-reactivity with HAZV NP, may also be explained by this crystal structure. Of the 12 CCHFV NP residues that interact with Affimer-NP (highlighted in orange in Fig 4D and 4E), 3 are not conserved in the HAZV NP (N399, L405, K336, highlighted in blue in Fig 4D and 4E), suggesting these changes are likely responsible for the differential recognition of CCHFV and HAZV NPs.

### Validation of Affimer-NP as recognition molecule in a sandwich ELISA

**Fig 5:**
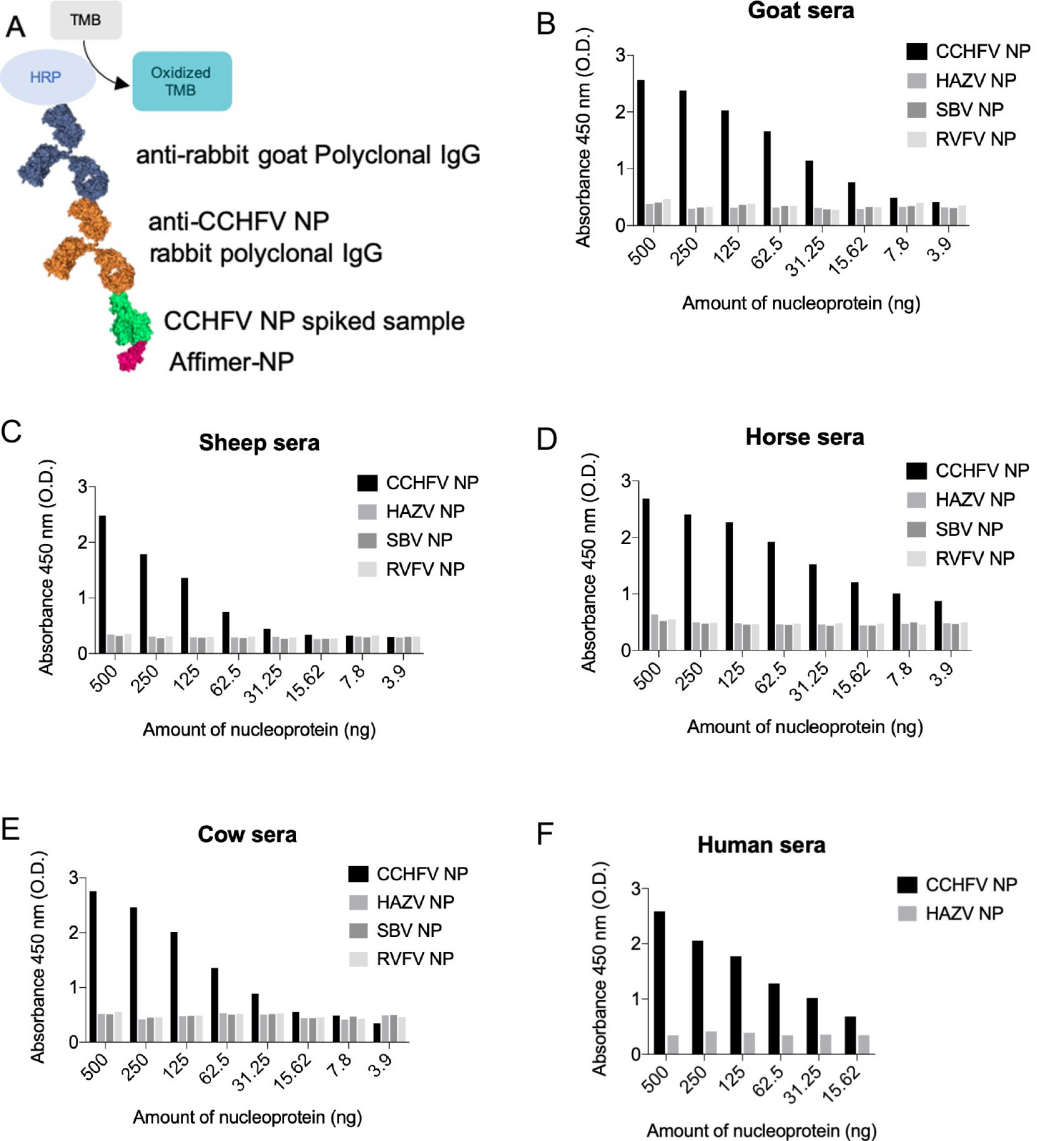
Sandwich ELISA for the detection of CCHFV NP. (A) Affimer-NP was used as capture molecule for CCHFV NP in spiked sera. Anti-CCHFV NP IgGs were used as detection molecule. Anti-rabbit goat polyclonal IgGs labelled with HRP were used to develop the colorimetric output using TMB as substrate. (B, C, D, E, F) ELISA results testing sera from animals (B-E) or human (F) spiked with CCHFV, HAZV, SBV or RVFV NPs.

The high affinity, specificity and binding kinetics of Affimer-NP for CCHFV NP suggested it could act as a recognition molecule for development of *in vitro* diagnostic tests. First, Affimer-NP was tested as a capture molecule in sandwich ELISA. Briefly, 96-well plates were coated with Affimer-NP and incubated with different concentrations of CCHFV NP diluted in animal or human sera. As negative controls, other bunyaviral NPs namely HAZV, Rift Valley fever virus (RVFV) and Schmallenberg virus (SBV) were also tested. Anti-CCHFV NP IgGs were used as primary recognition molecule, and a secondary anti-rabbit antibody labelled with HRP was used to provide a colorimetric output (Fig 5A). Concentration of Affimer-NP, primary and secondary antibodies were optimized to obtain the best signal-to-background ratios.

The Affimer-NP based sandwich ELISA detected CCHFV NP in spiked sera from horse, goat, sheep, cow and human sources. The limit of detection (LOD) varied for the different sera samples, ranging from 31.25 ng/well (sheep) to less than 3.9 ng/well (horse). No cross-reactivity was shown when testing the negative control NPs of HAZV, RVFV and SBV (Fig 5B–5F).

### Validation of Affimer-NP as recognition molecule in a lateral flow assay

**Fig 6:**
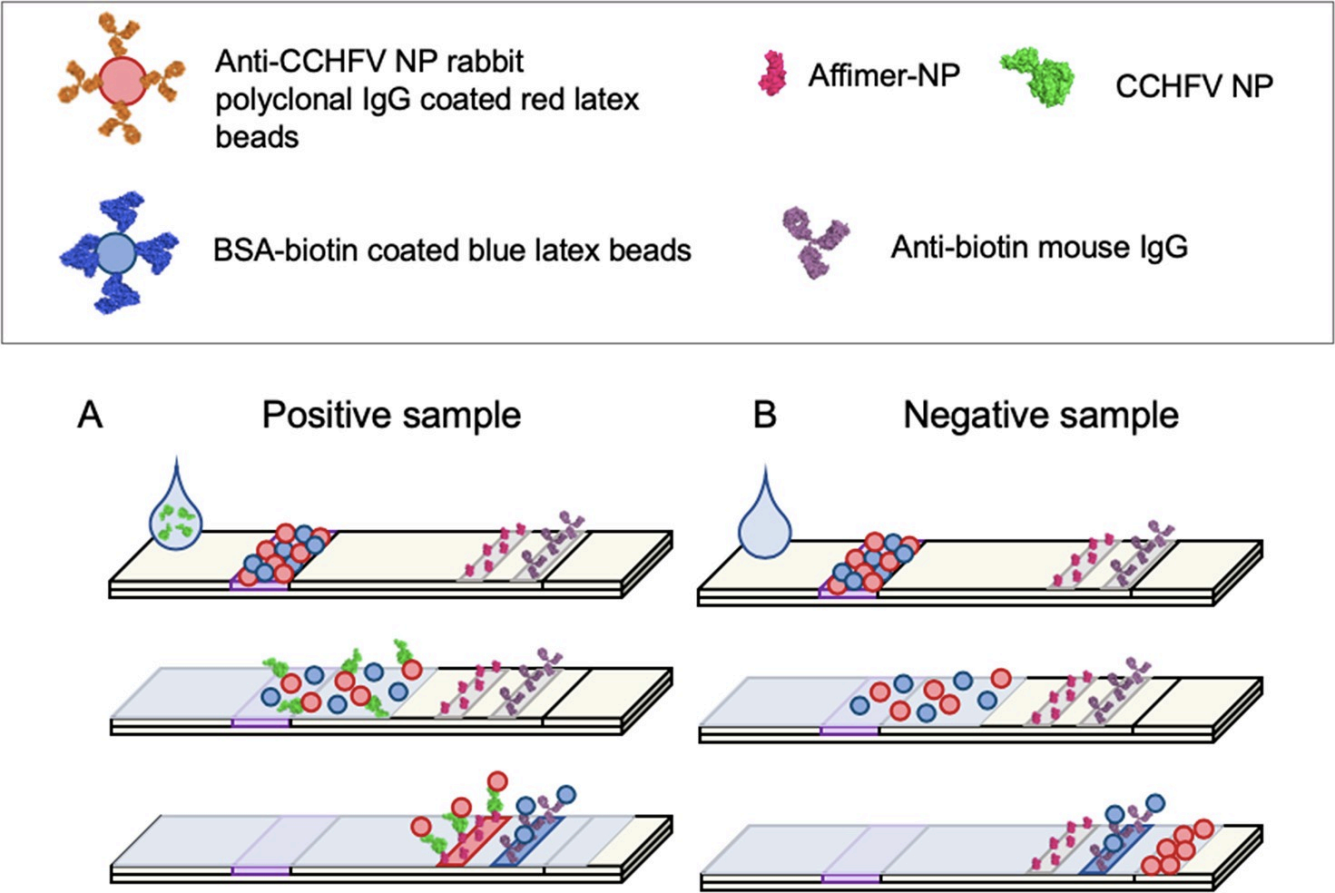
Development of a LFA for the detection of CCHFV NP using Affimer-NP as detection molecule. (A, B) Schematic of the LFA. Affimer-NP was used as detection molecule on the membrane of the LFA strip and anti-CCHFV NP polyclonal rabbit IgGs were used as capture antibody for the coating of red latex beads. Control anti-biotin mouse IgGs were used for the control line in the membrane together with biotin-BSA for the coating of blue latex beads. If a CCHFV NP spiked sample (A) is applied to the strip, the CCHFV NP binds to the antibody in the red beads and it is subsequently captured by the Affimer-NP in the membrane, resulting in a red test line. In the case of a negative sample (B), there is no CCHFV NP present and the red latex beads migrate until the end of the strip, resulting in an uncolored test line. In both cases, the BSA-biotin coated blue latex beads bind to the anti-biotin mouse IgG on the membrane, resulting in a blue control line.

**Fig 7:**
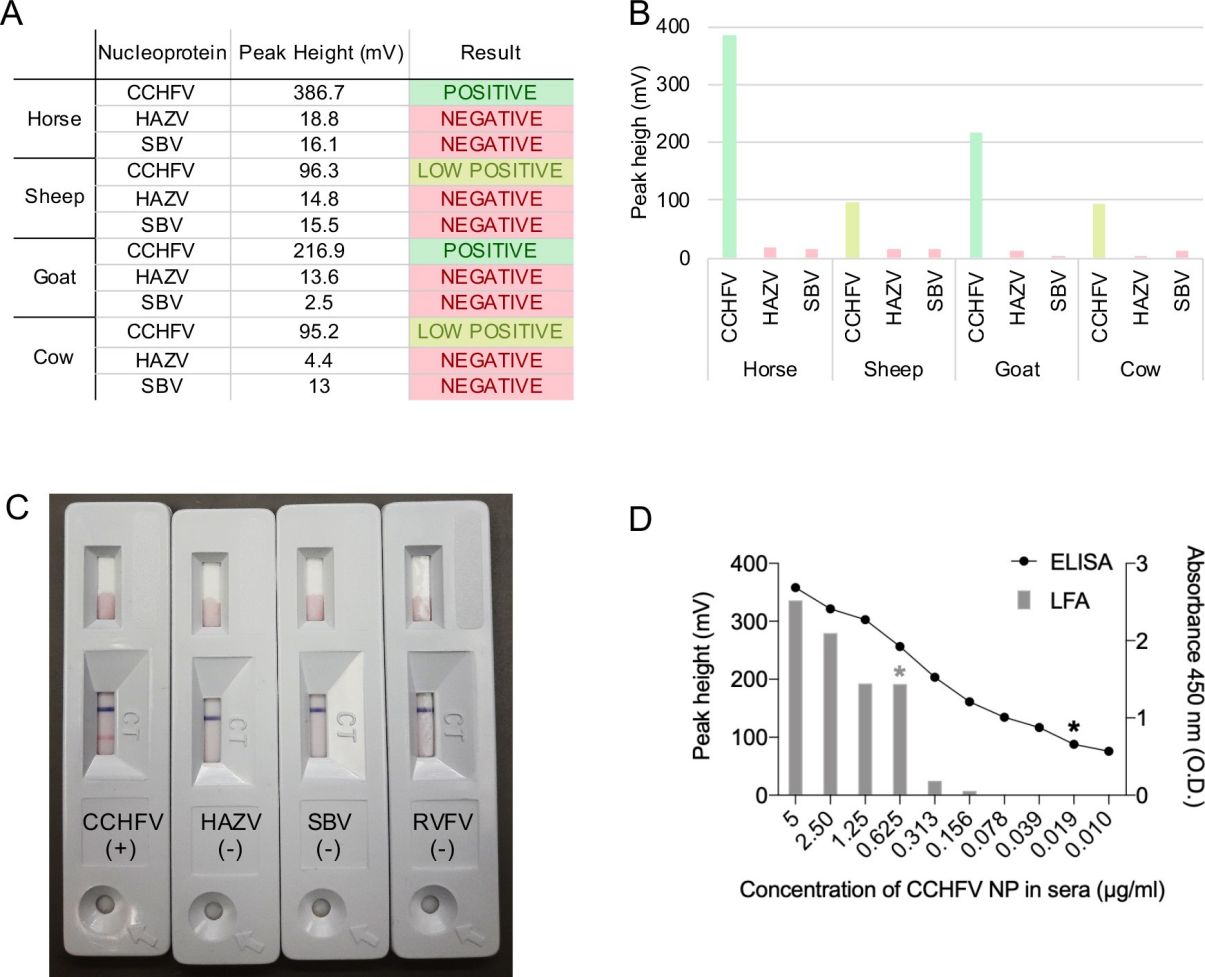
Affimer-NP based LFA results and comparison with ELISA. (A) LFA positive line intensity measured after applying animal sera spiked with CCHFV, HAZV or SBV NP (120 ng NP/strip). (B) Histogram representation of data in (A). (C) LFAs 10 min after application of horse sera samples spiked with CCHFV, HAZV, SBV or RVFV NP. (D) Comparison of performance between ELISA and LFAs using horse sera spiked with CCHFV NP. Primary axes correspond to LFAs and secondary axes to ELISA. Limits of detection are highlighted with an asterisk.

Next, we tested the suitability of Affimer-NP for development of a lateral flow assay (LFA) (Fig 6). Briefly, red latex beads were coated with anti-CCHFV NP IgGs and Affimer-NP was deposited on the LFA membrane. Concentration of antibody and Affimer-NP were optimized to obtain the strongest signal in the test line. For the control line, BSA-biotin was conjugated to blue latex particles and an anti-biotin IgG monoclonal antibody was dispensed in the membrane. The size of the latex beads was monitored by dynamic light scattering to avoid use of aggregated particles (S7 Fig). Spiked samples were prepared using different concentrations of CCHFV NP diluted in either animal or human sera, with samples applied to strips followed by addition of running buffer. NPs of other bunyaviruses (HAZV, RVFV and SBV) were also tested as negative controls. The strength of the control line was evaluated by eye (Fig 7C) and quantified using an ESE Quant Lateral Flow reader (Fig 7A and 7B).

The Affimer-NP based LFA detected recombinant CCHFV NP in spiked sera from horse, goat, sheep, cow and human. No cross-reactivity was shown when testing these sera spiked with negative control NPs from other bunyaviruses (Fig 6A–6C). At least 10 negative field sera samples from each animal and from humans were tested, and none gave false positives, demonstrating a 100% specificity of the LFAs for CCHFV NP under these conditions.

The LOD of both assays was compared using horse sera spiked with different concentrations of CCHFV NP (Fig 7D). The sandwich ELISA detected down to 1.5 ng of recombinant CCHFV NP/well (0.015 μg/mL), whereas the LOD of CCHFV NP by LFA was 15 ng/strip (0.75 μg/mL).

## Discussion

CCHFV-mediated disease is an important public health issue and an urgent need for accelerated research in its diagnosis and treatment has been declared [11]. Here, we generated and characterised a novel CCHFV NP specific binding molecule, Affimer-NP, that exhibited an inhibitory effect in CCHFV gene expression and possesses potential for use in diagnostic applications.

Affimers for more than 100 targets have been previously described in the literature with high specificity and low nanomolar affinities [26]. Likewise, Affimer-NP presents high specificity for CCHFV NP, lacking cross-reactivity with its close homologue HAZV NP, and exhibiting high affinity with a nanomolar K_D_.

The secondary structure and melting temperatures obtained for Affimer-NP and CCHFV NP are consistent with previously published data [7,24,26]. The high Tm of Affimer-NP indicates high thermostability, which is a characteristic feature of Affimer reagents [26]. The mixture of Affimer-NP and CCHFV NP presented a higher Tm than CCHFV NP, suggesting that the binding of Affimer-NP delays the melting of alpha-helical structures in the nucleoprotein inducing its thermostabilization.

A specific interference of Affimer-NP in the RNA-binding function of CCHFV NP and CCHFV gene expression was demonstrated in the context of a newly-developed CCHFV mini-genome system. While this system lacks the authenticity of a CCHFV infection, it allows specific examination of the effect of Affimer-NP at the stage of RNA synthesis, highly relevant to its NP-binding properties. While further investigations of Affimer-NP inhibitory properties need to be performed using infectious virus and animal models, our findings suggest a potential antiviral effect of this molecule. The antiviral drug ribavirin is the only currently available therapeutic agent for the treatment of CCHFV infections, but its mechanism of action remains to be elucidated and case–control studies have not been conducted to date [23]. The use of Affimers for therapeutic purposes has not been yet fully investigated, but their small size, and high stability and solubility make them attractive candidates for therapeutic applications [26].

A better understanding of the Affimer-NP/CCHFV NP interaction was achieved by the elucidation of the crystal structure of the complex. The variable loops of Affimer-NP bind to the globular domain of CCHFV NP with interaction sites including residues previously predicted to participate in both NP-RNA interactions and oligomerization, suggesting a possible interference of Affimer-NP in these two fundamental NP functions. The specificity of Affimer-NP for CCHFV NP and the lack of cross-reactivity with HAZV NP can also be explained by the crystal structure of the complex. Affimers have shown high selectivity in previous studies, being able to differentiate even between analogues of small molecules [27]. From the predominant 12 residues involved in the interaction contacts between Affimer-NP and CCHFV NP, only 3 are not conserved in HAZV NP, seemingly being enough to prevent its recognition by Affimer-NP.

The high affinity, specificity and stability of Affimer-NP suggested it represents a promising candidate for detection of CCHFV NP in patient sera as a diagnostic assay. Accurate diagnosis of CCHFV can be achieved when molecular methods are combined with antibody detection in serial serum samples [31], but the performance of these techniques is not always suitable in remote areas or in low-resource settings, creating the need of a point-of-care test for early diagnosis. Affimer-NP showed an efficient performance as a capture molecule in a sandwich ELISA format, detecting down to 1.5 ng of recombinant CCHFV NP per 100 μL of human and animal sera. A previous study has shown that an antibody-based antigen-capture ELISA with a LOD of 2 ng of recombinant CCHFV NP per 100 μL was suitable for the detection of native CCHFV in acute sera of CCHFV-infected patients [32]. According to these observations, the Affimer-NP based sandwich ELISA has a similar LOD to an antibody-based ELISA, and consequently is likely to be capable of detecting native CCHFV NP in acute patient samples, but this important validation remains to be experimentally demonstrated in future work.

The Affimer-NP based LFAs presented in this study require minimal specimen processing, have time to results of minutes and are suitable for field testing and low-infrastructure settings. The high thermostability of Affimer-NP allows its immobilization into the LFA membrane, which involves a baking step at 45°C, and suggests such devices will be robust in the field. This is a remarkable advantage of Affimer reagents, particularly relevant in low-resource settings where refrigerated storage of diagnostic tests may not be possible [33]. The use of Affimers as detection molecules also provides additional benefits due to their low production cost and consistent batch uniformity. In our hands, these tests present 100% specificity for CCHFV NP and their LOD is 15 ng of recombinant CCHFV NP/strip. There are no previous studies describing the LOD needed for LFA devices to successfully detect CCHFV NP in acute patient samples, nor reference antigen preparation standards for hemorrhagic fever viral NPs [34]. But different types of point-of-care diagnostic tests have shown the ability to detect hemorrhagic fever viral NPs in virus isolates and patient samples, such is the case for Lassa virus [35], Ebola virus [36] or RVFV [37]. In order to determine if the sensitivity of our current device is sufficient to detect CCHFV NP in real patient samples, it will need to be experimentally tested and optimized using patient samples under high-level biosafety containment. Nonetheless, the results presented in this study serve as proof of concept for the use of Affimers in LFAs and represent the first prototype point-of-care diagnostic test for the detection of recombinant CCHFV NP in sera described to date. These findings represent a possible starting point for the future development of Affimer-based diagnostic assays for the detection of CCHFV NP, contributing to the preparedness for potential future outbreak scenarios.

## Supporting information

S1 Fig Anti-CCHFV NP Affimer hits. (A) Alignment of the Affimer ORF amino acid sequences corresponding to the 7 Affimer hits obtained in the CCHFV NP Affimer screening after their subcloning into a pET11a vector. Variable loops (Loop 1 and Loop 2) and the C-terminal cysteine and 8xHis tail are indicated. (B) Different amounts (1, 0.1 or 0.01 μL) of biotinylated Affimers (0.5 mg/mL) were incubated with streptavidin-HRP and TMB.
(TIF)

S2 Fig Anti-CCHFV NP IgGs cross-react with HAZV NP and vice versa. (A) SDS-PAGE and western blot analysis of recombinant CCHFV NP and HAZV NP using anti-CCHFV NP as primary antibody. (B) SDS-PAGE and western blot analysis of recombinant CCHFV NP and HAZV NP using anti-HAZV NP as primary antibody.
(TIF)

S3 Fig SPR data fitting. (A) Fitting of the SPR sensogram corresponding to the binding of CCHFV NP (10 nM) and Affimer-NP to a Lagmuir 1:1 binding model. (B) Association (k_on_), dissociation (k_off_) and affinity (K_D_) constants and Chi^2^ value obtained from the fit curve in (A).
(TIF)

S4 Fig Prediction of secondary structure elements. (A, B, C) Percentage of alpha-helices, beta-sheets, turns and other secondary structure elements of Affimer-NP (A), CCHFV NP (B) and the complex (C) at 20°C. (D) Normalized predicted alpha-helical content of CCHFV NP and Affimer-NP/CCHFV NP complex at different temperatures (20°C to 90°C).
(TIF)

S5 Fig Direct binding fluorescence anisotropy analyses. (A, B) RNA-binding of CCHFV NP to 27mer (A) or 48mer (B) synthetic RNA molecules. Data in (A) and (B) are presented as mean ±SD (n = 3 replicates) and are fitted to a nonlinear regression curve.
(TIF)

S6 Fig Purification of native Affimer-NP and Affimer-NP/CCHFV NP complex. (A) SDS-PAGE and Coomassie staining analysis of the different fractions obtained during the first Ni^2+^-NTA affinity chromatography. (B) SDS-PAGE and Coomassie staining analysis of the different fractions obtained during the cleavage of the 6xhis-SUMO tag and a second Ni^2+^-NTA affinity chromatography. (C) Chromatogram of the size exclusion chromatography of Affimer-NP after the second Ni^2+^-NTA affinity chromatography. (D) SDS-PAGE analysis and Coomassie staining of the size exclusion chromatography fractions containing native Affimer-NP. (E) Chromatogram of the size exclusion chromatography of Affimer-NP/CCHFV NP complex. (F) SDS-PAGE analysis and Coomassie staining of the size exclusion chromatography fractions containing the complex.
(TIF)

S7 Fig Dynamic light scattering analyses of latex beads. Size distribution of beads functionalized with anti-CCHFV NP IgGs (black) and a mix of latex beads functionalized with anti-CCHFV NP IgGs and control biotin-BSA beads (grey).
(TIF)

S1 Table X-Ray Crystallography data collection and refinement statistics. (DOCX)
