# Supplementary figures and images for: Characterization and applications of a Crimean-Congo hemorrhagic fever virus nucleoprotein-specific Affimer: Inhibitory effects in viral replication and development of colorimetric diagnostic tests
Source: PLoS Negl Trop Dis. 2020 Jun 3;14(6):e0008364. doi: 10.1371/journal.pntd.0008364 (PMC7295242; doi:10.1371/journal.pntd.0008364)

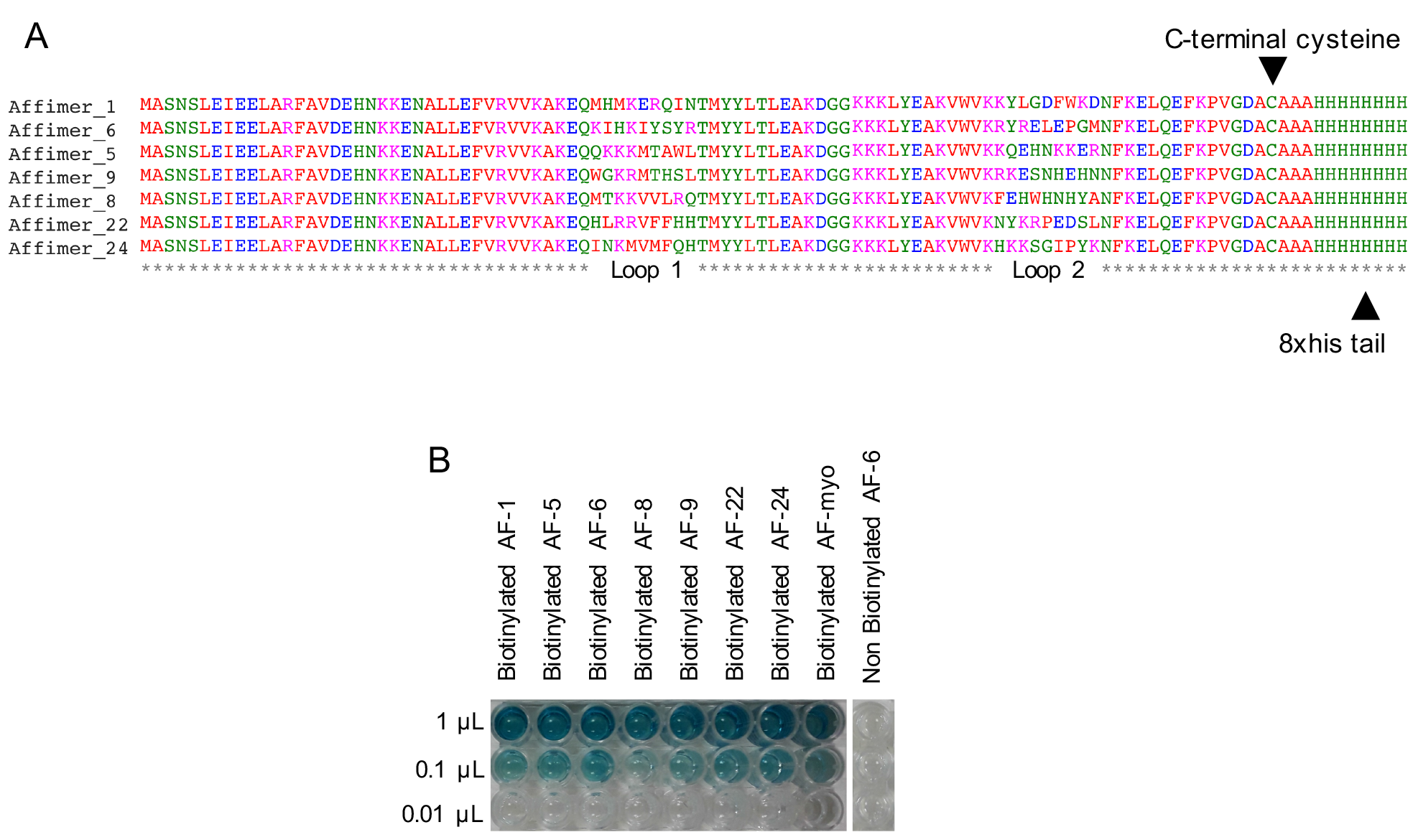

Supplement: S1 Fig — (A) Alignment of the Affimer ORF amino acid sequences corresponding to the 7 Affimer hits obtained in the CCHFV NP Affimer screening after their subcloning into a pET11a vector. Variable loops (Loop 1 and Loop 2) and the C-terminal cysteine and 8xHis tail are indicated. (B) Different amounts (1, 0.1 or 0.01 μL) of biotinylated Affimers (0.5 mg/mL) were incubated with streptavidin-HRP and TMB. (TIF) [file pntd.0008364.s001.tif]

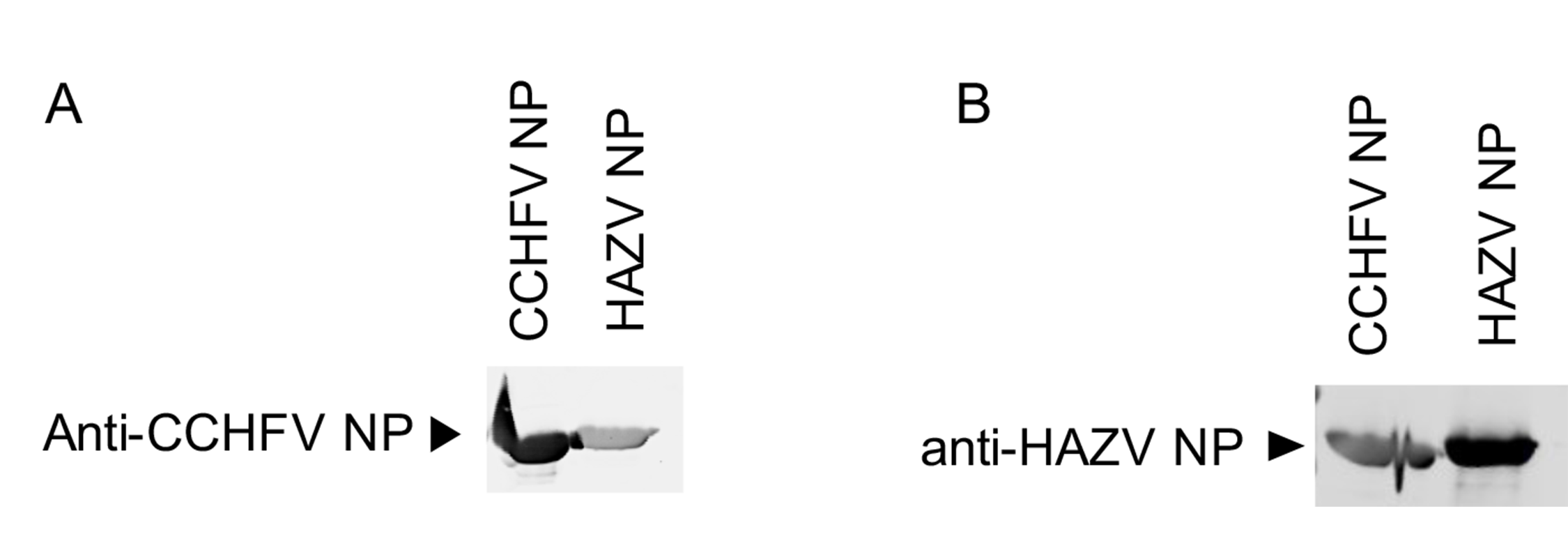

Supplement: S2 Fig — (A) SDS-PAGE and western blot analysis of recombinant CCHFV NP and HAZV NP using anti-CCHFV NP as primary antibody. (B) SDS-PAGE and western blot analysis of recombinant CCHFV NP and HAZV NP using anti-HAZV NP as primary antibody. (TIF) [file pntd.0008364.s002.tif]

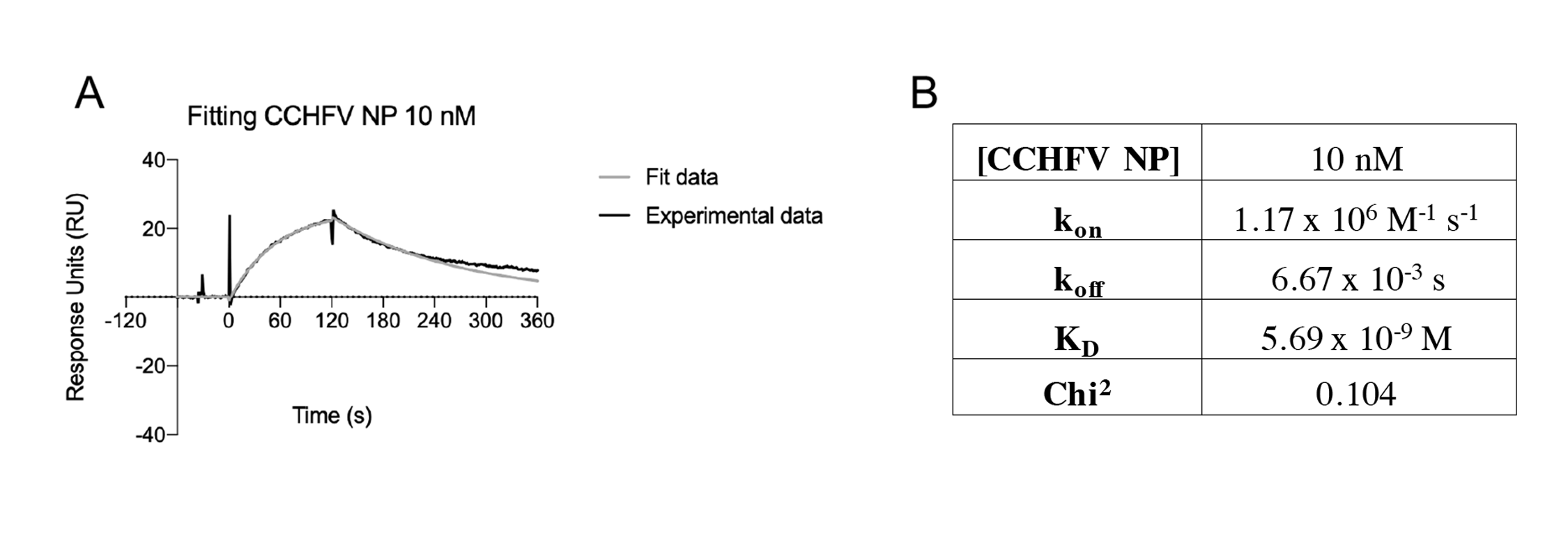

Supplement: S3 Fig — (A) Fitting of the SPR sensogram corresponding to the binding of CCHFV NP (10 nM) and Affimer-NP to a Lagmuir 1:1 binding model. (B) Association (kon), dissociation (koff) and affinity (KD) constants and Chi2 value obtained from the fit curve in (A). (TIF) [file pntd.0008364.s003.tif]

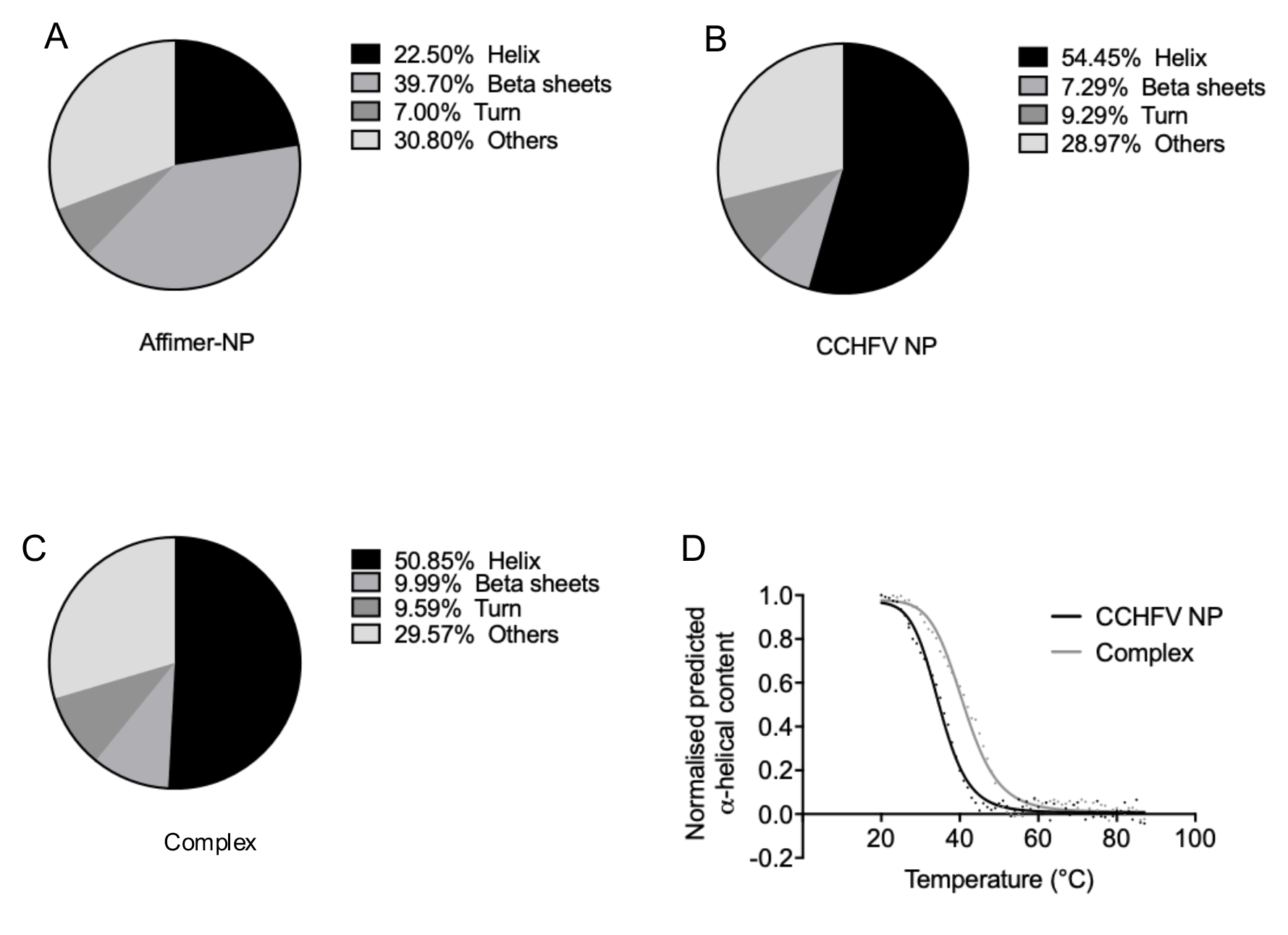

Supplement: S4 Fig — (A, B, C) Percentage of alpha-helices, beta-sheets, turns and other secondary structure elements of Affimer-NP (A), CCHFV NP (B) and the complex (C) at 20°C. (D) Normalized predicted alpha-helical content of CCHFV NP and Affimer-NP/CCHFV NP complex at different temperatures (20°C to 90°C). (TIF) [file pntd.0008364.s004.tif]

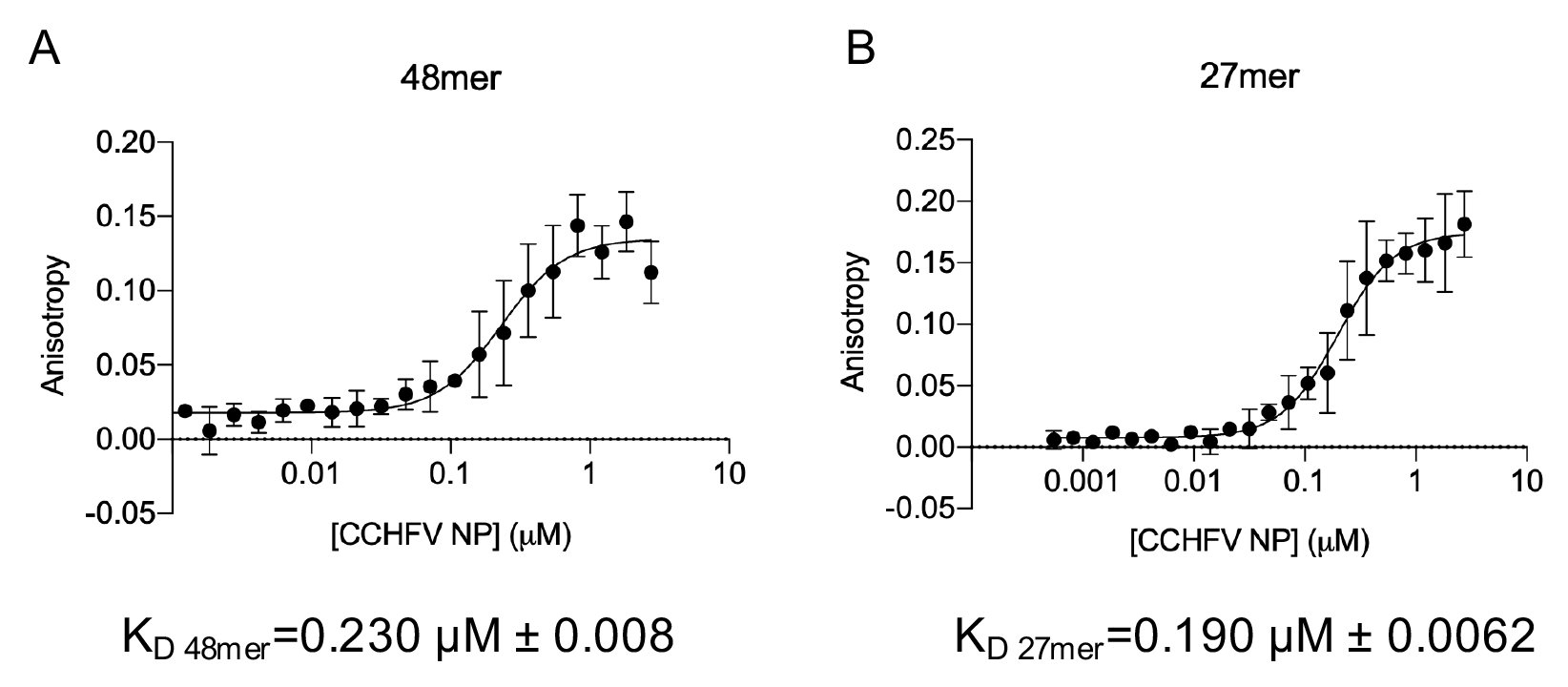

Supplement: S5 Fig — (A, B) RNA-binding of CCHFV NP to 27mer (A) or 48mer (B) synthetic RNA molecules. Data in (A) and (B) are presented as mean ±SD (n = 3 replicates) and are fitted to a nonlinear regression curve. (TIF) [file pntd.0008364.s005.tif]

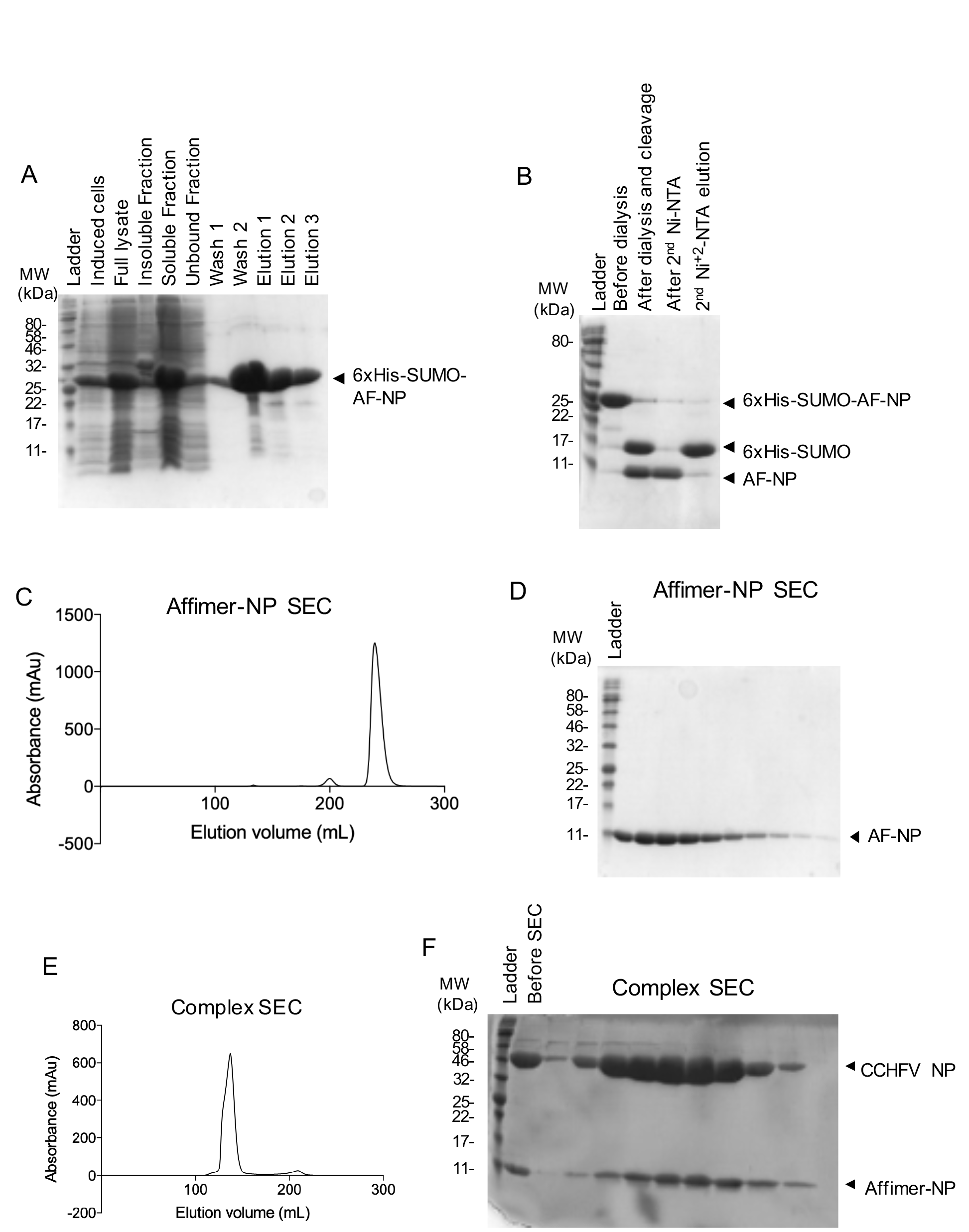

Supplement: S6 Fig — (A) SDS-PAGE and Coomassie staining analysis of the different fractions obtained during the first Ni2+-NTA affinity chromatography. (B) SDS-PAGE and Coomassie staining analysis of the different fractions obtained during the cleavage of the 6xhis-SUMO tag and a second Ni2+-NTA affinity chromatography. (C) Chromatogram of the size exclusion chromatography of Affimer-NP after the second Ni2+-NTA affinity chromatography. (D) SDS-PAGE analysis and Coomassie staining of the size exclusion chromatography fractions containing native Affimer-NP. (E) Chromatogram of the size exclusion chromatography of Affimer-NP/CCHFV NP complex. (F) SDS-PAGE analysis and Coomassie staining of the size exclusion chromatography fractions containing the complex. (TIF) [file pntd.0008364.s006.tif]

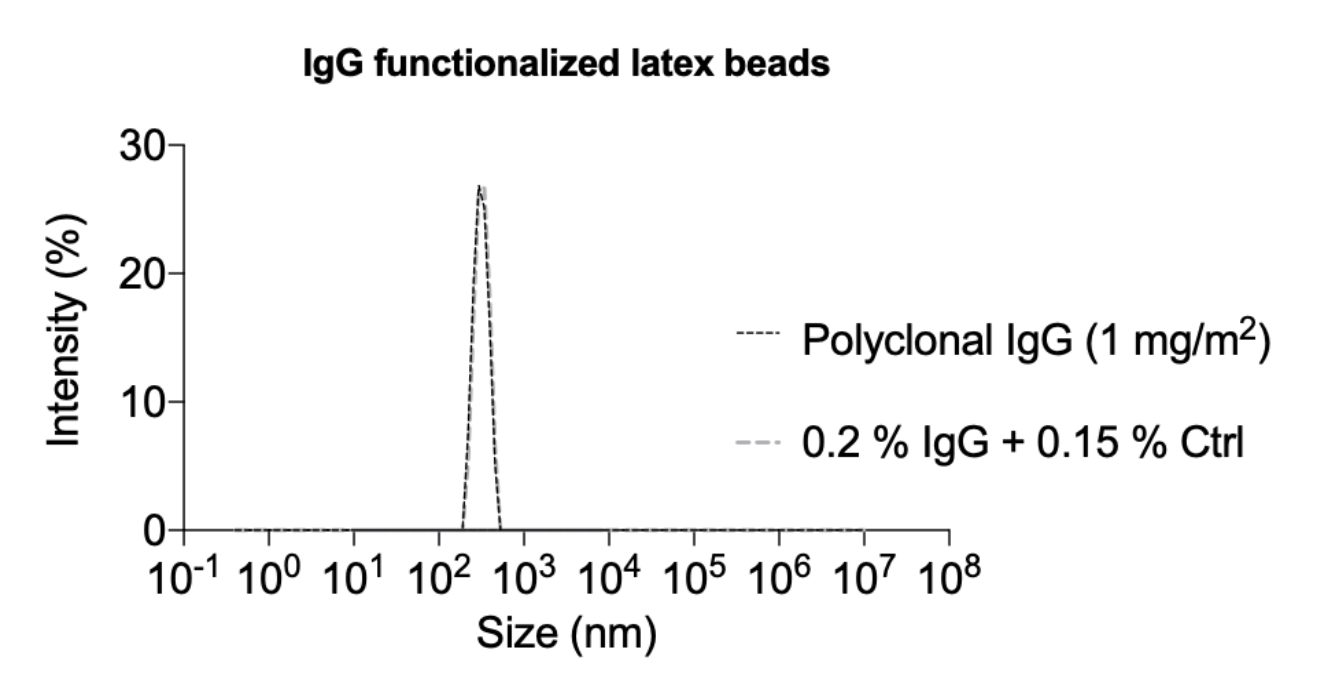

Supplement: S7 Fig — Size distribution of beads functionalized with anti-CCHFV NP IgGs (black) and a mix of latex beads functionalized with anti-CCHFV NP IgGs and control biotin-BSA beads (grey). (TIF) [file pntd.0008364.s007.tif]
