# Supplementary material for: Characterization and applications of a Crimean-Congo hemorrhagic fever virus nucleoprotein-specific Affimer: Inhibitory effects in viral replication and development of colorimetric diagnostic tests
Source: PLoS Negl Trop Dis. 2020 Jun 3;14(6):e0008364. doi: 10.1371/journal.pntd.0008364 (PMC7295242; doi:10.1371/journal.pntd.0008364)
Supplement: S1 Table — (DOCX) [file pntd.0008364.s008.docx]

| **Data collection** | | | |
| --- | --- | --- | --- |
| Unit cell parameters | a = 90.33 b = 73.98 c = 95.76 (Å)  α = 90.00° β = 100.92° γ = 90.00° | | |
| Space group | P 2_1_ | | |
|  | Overall | Inner | Outer |
| Low resolution limit | 94.03 | 94.03 | 2.71 |
| High resolution limit | 2.60 | 9.00 | 2.6 |
| Number unique reflections | 26028 | 965 | 126 |
| Mean((I)/sd(I)) | 10.3 | 23.3 | 1.1 |
| Half-set correlation CC(1/2) | 0.998 | 0.998 | 0.219 |
| Completeness % | 67.1 | 98.9 | 2.6 |
| Overall, resolution estimate | 2.84Å | | |
| **Refinement** | | | |
| R-work | 0.2331 | | |
| R-free | 0.2665 | | |
| RMS(angles) | 0.96 | | |
| RMS(bonds) | 0.005 | | |
| Ramachandran outliers | 0.6% | | |
| Ramachandran favored | 93.1% | | |
| Rotamer outliers | 5.2% | | |
| C-beta outliers | 0 | | |
| Molprobity Clashscore | 4.02 | | |
| Molprobity Overall score | 2.17 | | |
